# Identifying aspects of palliative and end-of-life care that are most important to people with lived experience and can be measured using routine data: a series of patient and public involvement workshops

**DOI:** 10.1186/s41687-026-01057-6

**Published:** 2026-04-18

**Authors:** Lesley E. Williamson, Georgina Macdonald, Joanna M. Davies, Rashmi Kumar, Margaret Ogden, Kamil Sterniczuk, Samina Begum, Mina Tanna, Peter May, Lorna K. Fraser, Fliss E. M. Murtagh, Irene J. Higginson, Katherine E. Sleeman

**Affiliations:** 1 Cicely Saunders Institute of Palliative Care, Policy & Rehabilitation, King’s College London, London, UK; 2 School of Medicine, Trinity College Dublin, Dublin, Ireland; 3 Wolfson Palliative Care Research Centre, Hull York Medical School, University of Hull, Hull, UK

**Keywords:** Public involvement, Routinely collected health data, Palliative care, End of life care, Quality of health care

## Abstract

**Background:**

We aimed to identify which aspects of palliative and end-of-life care are most important to people with lived experience of life-limiting conditions and can be measured using routine data currently available in the UK.

**Methods:**

Informed by a rapid umbrella scoping review and search of routine data available, we ran three online Patient and Public Involvement (PPI) workshops. 12–14 people from diverse backgrounds with experience of palliative and end-of-life care attended each workshop.

**Results:**

We identified nine domains of care that are important to people with lived experience of life-limiting conditions. Within four domains, there were ten aspects of care that are directly or indirectly measurable using available routine data in the UK. These included continuity of care, avoiding emergency care, early palliative care, control of physical symptoms, including pain, and achieving preferred place of death. Important aspects of care not measurable in current routine data related to domains of emotional, family and spiritual wellbeing, communication, trust and expertise.

**Conclusions:**

Research using routine data in palliative and end-of-life care should focus on the areas identified as important to those with lived experiences. Incorporating patient-reported outcome measures into clinical records would help to address gaps in aspects of care not currently measurable using routine data.

**Supplementary Information:**

The online version contains supplementary material available at 10.1186/s41687-026-01057-6.

## Abstract (plain language summary)

**1. Why is this study needed?**

Routine data, such as data from electronic patient records, is a powerful resource for researchers. It is recommended that researchers involve people with lived experience when analysing routine data.

**2. What is the key problem/issue/question this manuscript addresses?**

Research using routine data may focus on aspects of palliative and end-of-life care that can be measured, rather than what is most meaningful to patients and carers. We aimed to identify aspects of care that can be measured and are meaningful.

**3. What is the main point of your study?**

Most aspects of care that are important to people with lived experience cannot be measured using routine data that is currently available. Including patient-reported outcomes in clinical records will help to address this gap.

**4. What are your main results and what do they mean?**

Ten aspects of care that were important to people with lived experience were measurable using routine data, including control of physical symptoms and avoiding emergency care. These aspects of care should be prioritised in research when using routine data. The findings also highlight that available routine data does not reflect the breadth of what is important to people, and we recommend including patient-reported outcomes in clinical records to help with this.

**Supplementary Information:**

The online version contains supplementary material available at 10.1186/s41687-026-01057-6.

## Background

High quality care towards the end of life is associated with improved outcomes for patients, caregivers and the health care system. For example, specialist palliative care is associated with improved quality of life, fewer symptoms, and less depression for people who are dying [1]. For carers, palliative care is associated with improved outcomes in bereavement [2]. Palliative care improves integration of care, as patients have fewer emergency hospital attendances near the end of life and are more likely to die in their own homes [3, 4]. Palliative care is therefore associated with reduction in health care costs, and savings are found to be highest for those with the most complex needs [5].

The ageing population means that the number of people dying with palliative care needs is expected to increase [6]. It is currently estimated that 1 in 3 people die with unmet palliative care needs [7]. Inequalities in aspects of palliative and end-of-life care are well documented, including associations between outcomes and socio-economic position [8], and ethnicity [9], which are widening over time [10]. Given this, without intervention, we are likely to see increasing unmet need towards the end of life, and worsening inequalities.

Routine data, such as electronic health record data, are generated by administrative and clinical processes and are widely used in health research [11]. With minimal burden for patients and families, routine data has marked value in palliative care research [11]. Routine data has advantages of whole-population coverage and reflects real-world processes [12], making routine data analysis particularly helpful for understanding and addressing inequalities. However, while research has explored meaningful outcomes for people approaching the end of life [13, 14], this work has not been mapped onto existing sources of routine data. In turn, research using routine data can tend to focus on what is easily measured, rather than what is most meaningful. While this may be at the area, service or individual level [11], many palliative and end-of-life care quality indicators are process measures and overlook social, psychological, cultural and spiritual aspects of care [15]. Few are based on patient or proxy reported outcomes [15], despite being considered gold standard for measuring quality of care [11].

It is recommended that the development and selection of outcomes in routine data is informed by patients and carers [16, 17]. While a complex topic, patients and others with lived experience can meaningfully engage in research using routine data [18–20], and in doing so, contribute to improving public trust and driving ethical governance [21]. Furthermore, engagement of people from minoritised communities in routine data research can inform efforts to reduce inequalities [19]. Therefore, we carried out patient and public involvement (PPI) workshops to identify which aspects of palliative and end-of-life care are most important to people with lived experience of life-limiting conditions and can be measured using routine data currently available in the UK.

## Methods

### Design and setting

From October 2023 to January 2024, we ran three three-hour online workshops with people with lived experience of life-limiting conditions. This was part of a larger research project exploring the use of routine data to examine and improve inequalities in end-of-life care [22].

To inform discussions, we conducted a rapid umbrella scoping review on aspects of palliative and end-of-life care that are important to patients and families (Additional file, A1). Review findings were synthesised and deductively categorised according to a pre-established framework comprising seven palliative care outcome domains based on international expert consensus [23]. One domain was inductively refined, and another was separated into two domains, based on the review findings. This resulted in an initial ‘evidence wheel’ comprising eight domains, which acted as a focus for workshop discussions.

**Fig. 1:**
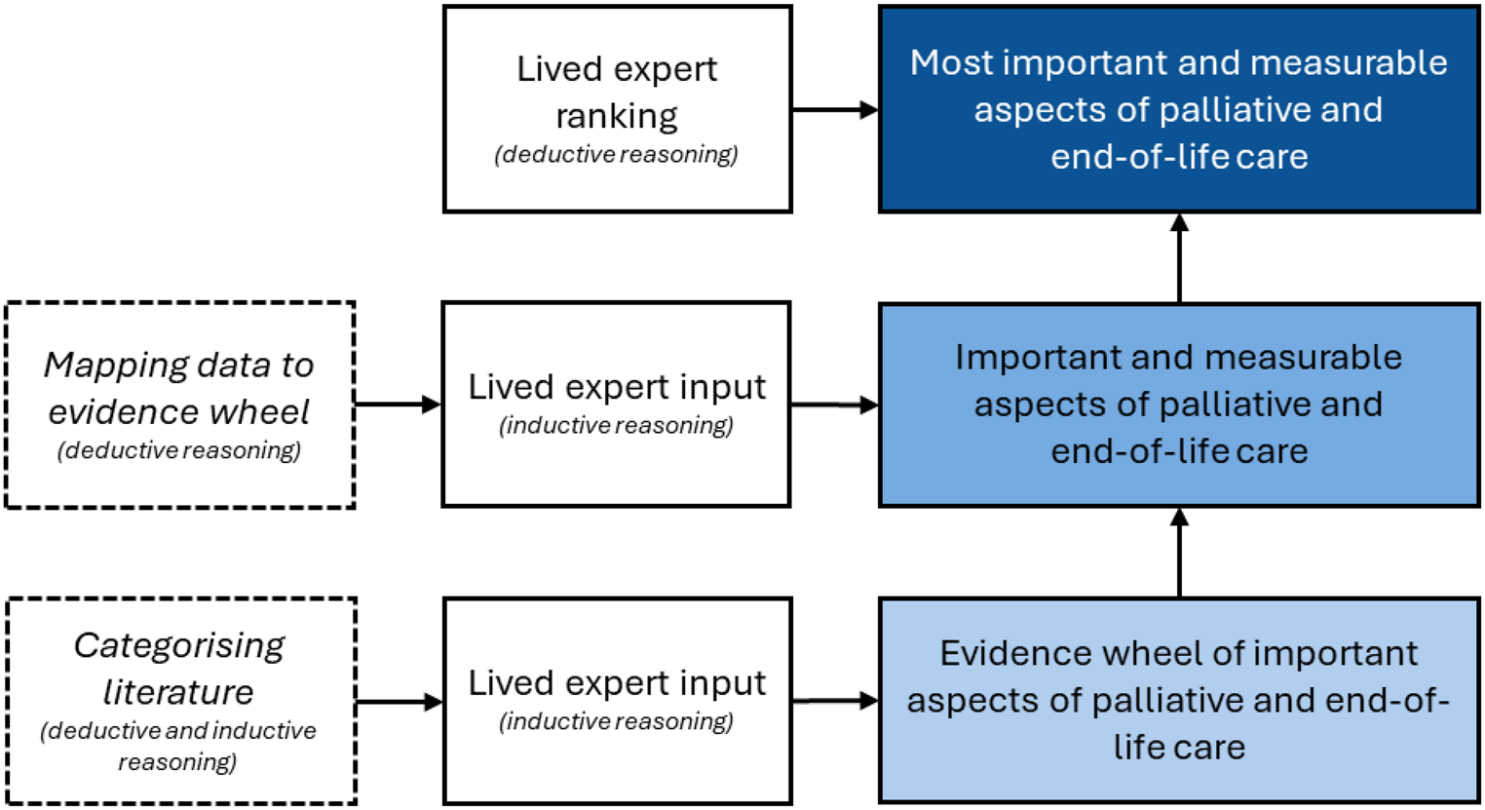
Schematic of how previous literature, currently available routine data and lived experience informed prioritisation and measurability

Workshop 1 explored important aspects of palliative and end-of-life care, from which we inductively refined the domains of the evidence wheel. Workshop 2 involved discussion of the important aspects of palliative and end-of-life care that can be measured using available routine data. To inform discussions, we presented findings of a desk-based search of health and social care population datasets available in the UK, mapped against relevant domains of the evidence wheel. Workshop 3 prioritised aspects of palliative and end-of-life care that are important to people with lived experience and are measurable using currently available routine data. See Fig. 1 for a schematic of this process.

### Workshop attendees

We advertised through established PPI forums: (1) Cicely Saunders Institute PPI forum and (2) University of Hull Involve forum, as well as snowballing. To ensure group diversity, we stated in the advert that we were particularly interested in hearing from people from minoritised ethnic groups and people with experience of financial difficulty. Interested individuals were invited to contact the research team directly by email. We screened expressions of interest to ensure a broad representation of different ethnicity, gender, socioeconomic position, geographical location, and diagnosis.

To promote inclusivity, attendees were emailed the workshop joining details, agenda, presentation slides and any supplementary material one week before, and again on the day of, the workshop. We also offered this material as hard copy to promote accessibility, though no attendee requested this.

### Data collection and analysis

We made notes of discussions during the workshops. With verbal agreement from attendees, we supplemented notes with recordings of workshop discussions and chat box comments. For consistency, LEW summarised key discussion points and cross-referenced these with the rapid umbrella review findings and available routine data. To enhance the credibility and trustworthiness of summaries, LEW checked these with attendees during the workshops. LEW also synthesised workshop summaries and discussed the summaries and their synthesis with the wider team to reflect on and interrogate assumptions.

We have included anonymised quotes in this paper to illustrate the key points identified, with documented retrospective agreement from each respective attendee [24].

### Reflexivity

We comprised a team of researchers with expertise in routine data analysis, patient-reported outcome measures, PPI, and clinical care of people approaching the end of life, and five members of a PPI Reference Group with lived experience of having relatives with terminal illness. Collectively, we designed the workshops, which were facilitated by KES and LEW and joined by all members of the PPI Reference Group, with researchers who were independent of the project taking notes. LEW led the rapid umbrella review, workshop discussion summaries and desk-based searching of datasets, with regular discussion and sense-checking with the wider research team and PPI Reference Group.

### Ethical consideration

We define PPI as the process of conducting research with or by members of the public, including patients and carers, to ensure relevance and meaning [20, 25]. As the workshops constituted the PPI component of research and not the research itself, no ethical approval was required [24]. However, we adopted an ethical approach to mitigate risk (Additional file, A2) [26]. For example, all quotations were anonymised, with any potentially identifiable details removed or altered to protect confidentiality. Every attendee was reimbursed up to £80.00 after each workshop, as per the National Institute for Health and Care Research (NIHR) public contributor payment policy [27].

We used the Guidance for Reporting Involvement of Patients and the Public (GRIPP2) to facilitate quality, transparent and consistent reporting (Additional file, A3) [28].

## Results

We received expressions of interest from 18 individuals; 14 took part (1 = did not respond; 1 = connectivity issues; 2 = competing commitments). The lived experience of attendees was diverse across sociodemographic characteristics (minoritised ethnicity = 9; financial hardship = 2; migration status = 1), health conditions (dementia = 3; cancer = 1; multimorbidity = 2; mental ill health = 2; physical disability = 2) and care settings (care homes = 1; hospices = 1; own homes = 2). All were current or bereaved carers of adults with a life-limiting condition.

### Workshop discussions

**Fig. 2:**
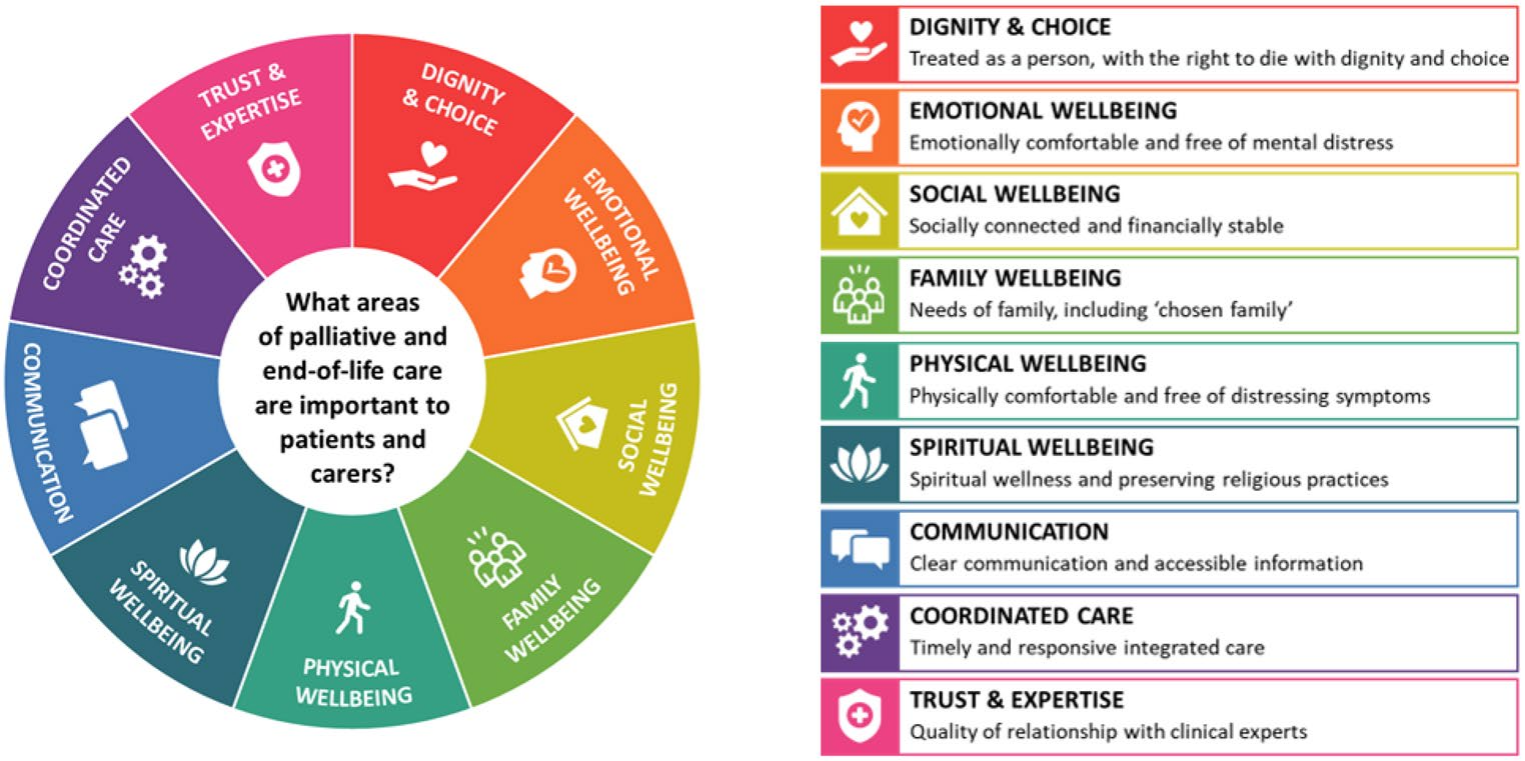
Evidence wheel of nine domains covering aspects of palliative and end-of-life care that are important to people affected by life-limiting conditions

Discussions in Workshop 1 built on findings of the umbrella review. This led to refinement of the evidence wheel, with one additional domain included and modifications to three others. The final ‘evidence wheel’ therefore comprised nine domains covering aspects of palliative and end-of-life care important to people affected by life-limiting conditions: Dignity and choice, Emotional wellbeing, Social wellbeing, Family wellbeing, Physical wellbeing, Spiritual wellbeing, Communication, Coordinated care, Trust and expertise (Fig. 2).

Ahead of Workshop 2, we identified aspects of care within each domain based on workshop 1 discussions and mapped these against routine data currently available in the UK. This resulted in ten, directly or indirectly measurable and important aspects of care, within four of the nine domains, which we presented and discussed with all 14 attendees in Workshop 2 (Table 1). These ten aspects of care were: Making an advance care plan, achieving preferred place of death, and discussing DNAR orders (under domain *Dignity and Choice*); Time spent in usual place of care (under domain *Social Wellbeing*); Physical symptoms under control, including pain, and physical symptoms under control during out-of-hours (under domain *Physical Wellbeing*); and Well-coordinated care, continuity of care, early palliative care, and avoiding emergency care (under domain *Coordinated Care*).

**Table 1:** Important and currently measurable aspects of palliative and end-of-life care

Aspects of care	Previous literature^a^	Workshop feedback^b^	Currently available routine data^c^	Group 1 ranking^d^	Group 2 ranking^d^
**Dignity & Choice**					
Having an identity and feeling like an individual	✓	✓			
Right to die with respect, dignity and privacy	✓	✓			
Making an advance care plan	✓	✓	Completed advance care plans	9	1
Acknowledging limited options for place of care and death	✓	✓			
Achieving preferred place of death		✓	Preferred and actual place of death	5	8
Discussing DNAR orders		✓	Recorded in Summary Plans	10	10
Not ‘written off’ as a dying patient		✓			
**Emotional Wellbeing**					
Feeling in control	✓	✓			
Being able to think clearly and make decisions	✓				
Being in a safe environment, without fear of discrimination	✓				
Being able to accept and prepare for death	✓				
Living in the present	✓				
Keeping a sense of normality	✓				
Not feeling like a burden	✓				
**Social Wellbeing**					
Surrounded by and spending time with loved ones	✓	✓			
Feeling connected with others and involved in activities	✓	✓			
Having finances in order	✓				
Being aware of free care and receiving financial support in good time	✓				
Living in a home that can accommodate specialist equipment / bed	✓				
Time spent in usual place of care	✓	✓	Hospital admissions and length of stay	2	7
Residential and respite settings suited to cultural and clinical needs		✓			
**Family Wellbeing**					
Involvement of family members in care (including care after death)	✓	✓			
Family having bereavement support, mementos, and time and space to grieve	✓	✓			
Knowing that family will have social, financial and housing security after death	✓	✓			
Family being allowed to be with and hold relative	✓				
Family emotional wellbeing being supported		✓			
Respite / “breathing space” for family		✓			
Needs and preferences of multigenerational families respected		✓			
**Physical Wellbeing**					
Physical symptoms under control, including pain	✓	✓	Medication prescribed and dispensed	6	6
Physical symptoms under control during out-of-hours	✓	✓	Medication prescribed and dispensed	7	9
Having nutritional needs and fluids met, and not feeling thirsty	✓				
Effectiveness of pain relief – balancing side effects so not drowsy / can engage with others		✓			
**Spiritual Wellbeing**					
Having spiritual and religious needs and prescribed responsibilities towards the end of life and after death recognised and supported	✓	✓			
Feeling peace and hope	✓				
Having purpose and meaning (including legacy)	✓				
**Communication**					
Having honest discussions, being listened to, having repeated explanations and access to interpreters and language aids	✓				
Being given all information to make informed decision		✓			
Being informed of what to expect and what can do to help promote comfort of dying relative		✓			
Having a family spokesperson or advocate		✓			
Understanding rights and knowing routes to complain		✓			
(Earlier) information about available services, including hospices		✓			
**Coordinated Care**					
Well-coordinated care	✓	✓	GP referral and specialist care contacts	8	3
Continuity of care	✓	✓	GP continuity score	3	2
Access to care tailored to need	✓	✓			
Early palliative care	✓	✓	End-of-life care registration	1	5
Avoiding emergency care	✓	✓	Emergency care use	4	4
Communication across and between different teams		✓			
Joined up services		✓			
Shared records between primary, secondary and social care, including palliative care		✓			
Clinical care integrated with culturally sensitive complementary therapies		✓			
**Trust & Expertise**					
Trust in healthcare professionals	✓	✓			
Staff and services that are culturally aware and inclusive	✓	✓			
Care that is compassionate, person-centred, and non-judgemental	✓				
Clinical expertise		✓			

Legend: ^a^ Pre-Workshop 1, ^b^ Workshop 1, ^c^ Pre-Workshop 2, ^d^ Workshop 3; DNAR = do not attempt resuscitation; GP=General Practitioner

Twelve of the original attendees were present at Workshop 3 (2 = competing commitments). Attendees were randomly allocated into two groups to collectively rank the important and measurable aspects of care in order of priority (1 = highest, 10 = lowest; Table 1).

#### Most important and measurable aspects of palliative and end-of-life care

Between the two groups, aspects of care that ranked first were ‘early palliative care’ and ‘making an advance care plan’, while ‘discussing DNAR (do not attempt resuscitation) orders’, was ranked tenth position by both groups.

Both groups ranked ‘avoiding emergency care’ in fourth position. Attendees discussed how emergency care puts “*both patient and carer under a lot of pressure*” (A7W1, chat), and that care following admission is too late:

*Following on from [emergency department attendance]*,* it was like going from one extreme to another; all of a sudden*,* I got a hospital bed at home…I’ve got the occupational therapist in… the doctor actually did home visits… it was all sort of a little too late sort of thing.* (A2W1)

Both groups ranked ‘control of physical symptoms, including pain’ in sixth position. Some attendees had emphatically discussed pain relief as important to relatives who can feel helpless and to patients whose pain can be all-consuming:

*…throughout those last six weeks*,* the most important thing to [him] was the amount of pain he was in*,* you know*,* to get rid of that pain…* (A11W1).

‘Continuity of care’ ranked similarly highly between the two groups, which was considered important to measure over time and across settings:

…*as time passes*,* I feel that you can drop off the radar… having that continuity of care*,* in my mind*,* heads off a crisis.*. (A7W3)

Although ranked first by one group, ‘making an advance care plan’ was ranked ninth by the other group. However, attendees broadly agreed that *“the physical act of making a care plan doesn’t guarantee anything*” (A5W3) and that measurement should include who completes the care plan, if plans are followed, and when they are updated:

…*it’s not just a matter of a tick-box if the advance care plan is in place or not*,* but it’s about the quality of the advance care plan*. (A4W3)

Other aspects of care that varied in rankings included ‘time spent in the usual place of care’. This was considered particularly important to people with dementia.

## Discussion

Through a series of PPI workshops, we identified ten aspects of palliative and end-of-life care that are important to people with lived experience and are measurable using routine data currently available in the UK. These included ‘continuity of care’, ‘avoiding emergency care’, and ‘control of physical symptoms including pain’. However, we also found that many aspects of care that are important to people with lived experience are not measurable using currently available routine data.

The use of emergency care can be measured directly using hospital data, or indirectly using data on service activity, such as primary care end-of-life care registration and palliative care referrals in relation to unplanned hospital use [29, 30]. The use of unplanned hospital care, including emergency department visits and hospital admissions near the end of life, is commonly measured in research and is used as a quality indicator in service development and policy [31]. Our workshops indicate public support for using these indicators as priority measures for research in palliative and end-of-life care.

Workshop attendees also discussed advance care planning, suggesting that data collection should reflect advance care planning as a process rather than one-off event. As individual preferences can change over time, advance care planning should be regularly updated and accessible across providers [32]. Digital advance care planning approaches, with real-time documentation of discussions, have potential to facilitate this if implementation is optimised [33]. This could offer opportunity to measure the number of advance care plan reviews, and the actors involved, which may provide researchers with a more nuanced view when analysing routine data.

It was striking that only four of the nine domains included aspects of care that can currently be measured in routine data. This reflects research that shows few quality indicators of palliative and end-of-life care include the social, cultural, spiritual and psychological aspects of care [15], which are fundamental for the provision of person-centred care [16]. It highlights a critical gap; that we are currently unable to assess many important aspects of care using routine data.

Workshop discussions suggested ways that routine data could be refined to capture more meaningful insights. A transformative step would be to incorporate patient-reported outcome measures within clinical records—for example, using the Integrated Palliative care Outcome Scale (IPOS). The IPOS family of measures includes outcomes related to spiritual wellbeing such as feeling at peace, psychological wellbeing such as feeling anxious, social wellbeing such as interacting with others, and communication such as receiving sufficient information. Our findings can inform patient-reported outcome measure selection, development and implementation, as additional items could include other important aspects of care, such as religious needs and prescribed responsibilities. Integration and systematic collection of patient-reported outcome measures into routine data would permit comparisons of care and inform decisions to improve care quality [34]. Given this, we reiterate calls for the routine collection of patient-reported outcome data [35], specifically in palliative and end-of-life care.

While we have identified aspects of palliative and end-of-life care that are important to people with lived experience and measurable using currently available routine data, we acknowledge that some of the aspects we identify are only indirectly measurable and therefore may not always reflect care quality. For example, the use of prescribed and dispensed medication data may not reflect administration of medication or symptom relief. Nevertheless, prescription data can provide useful insights into the quality [36], and equality [37], of end-of-life care. Linking clinical record data, such as prescribing data, to patient-reported outcome data would bring further benefits for clinical and policy decisions [38]. Future initiatives to expand routine data resources, and mitigate the practical and methodological challenges of collecting and using patient-reported outcome data [38], must continue to involve patients and the public to ensure alignment with what is important as well as what is measurable [17].

### Critical reflection of PPI

We found the that using targeted advertisement, word-of-mouth, and taking steer from the PPI reference group helped to ensure we recruited workshop attendees with different backgrounds, experiences, and interests. This diversity was essential to inform original discussion and identify areas of inequality. Involvement of the same attendees across all three workshops ensured continuity, and informed the incremental nature of feedback, which enhanced analysis. Drawing on previous literature, the process of ongoing dialogue and sharing time and experiences may have helped to nurture trust between attendees and with researchers [39], facilitating transparency and depth of discussion.

While our targeted advertisement on established PPI forums and snowballing helped with diversity of workshop attendees, this may have excluded people who do not frequently engage in research. To mitigate this, it may have been helpful to additionally advertise through local community providers with established trusting relationships with people, especially those from marginalised communities. As mediators and gatekeepers, local providers can offer a means of connecting researchers with minoritised groups [40], and empowering them to engage in research.

Cognisant of potential differences in language and literacy among workshop attendees, we created an ‘evidence wheel’, optimising the visual representation of evidence to promote engagement and accessibility [41]. Although the format of the evidence wheel diluted the more detailed findings of the review, for the purpose of informing workshop discussions, we believe this was a useful and necessary step to optimise equity.

### Strengths and limitations

Workshop discussions were strengthened by building on a scoping review of existing literature, by involving the same people across all workshops, and by including people with diverse intersecting characteristics and experiences. Holding workshops online reduced geographical barriers to participating, though this may have limited engagement from people with poor digital access.

A limitation of the project was the lack of systematic approach to identifying available datasets and mapping data to workshop discussions. While there is a risk that some data resources were not identified or adequately mapped against important areas of care, we attempted to mitigate this by sense-checking findings with the wider project team who were able to advise on important gaps.

Another limitation is the lack of representation of people living with life-limiting illness, as we were only successful in recruiting current and bereaved carers of people with life-limiting illness. Family carers play a key role in the provision of palliative and end-of-life care as members of the care team [42, 43], and thus have insight into care needs, processes and outcomes. However, we acknowledge that people with life-limiting conditions will also have valuable and unique insights and further efforts to examine meaningful outcomes in routine data must prioritise their involvement.

While included in our eligibility criteria, we did not have any parents with experience of children’s palliative and end-of-life care services at the workshops. The prevalence of life-limiting conditions among children is greatest in areas of deprivation and ethnically diverse populations, and is expected to increase over the next decade [44]. Future work that examines what is meaningful and measurable from the perspectives of children and/or parents with experience of children’s palliative and end-of-life care is necessary.

## Conclusions

We identify ten priority areas, including continuity of care, avoiding emergency care, and control of physical symptoms, including pain, which should be reported in research using routine data in palliative and end-of-life care, as they reflect the priorities of those with lived experience. However, we also identify several areas of care that are not measurable in currently available routine data. Incorporating patient-reported outcome assessments in clinical records would be a step towards addressing this critical gap.

## Supplementary Information

Below is the link to the electronic supplementary material.

**Supplementary Material 1**: Additional file, A1

**Supplementary Material 2**: Additional file, A2

**Supplementary Material 3**: Additional file, A3

## Data Availability

Data sharing is not applicable to this article as no datasets were generated or analysed during the current study.

## Abbreviations

PPI: Patient and Public Involvement
NIHR: National Institute for Health and Care Research
DNAR: Do Not Attempt Resuscitation
GP: General Practitioner
