# Supplementary material for: Identifying aspects of palliative and end-of-life care that are most important to people with lived experience and can be measured using routine data: a series of patient and public involvement workshops
Source: J Patient Rep Outcomes. 2026 Apr 18;10:120. doi: 10.1186/s41687-026-01057-6 (PMC13369061; doi:10.1186/s41687-026-01057-6)
Supplement: Supplementary file 1 — Supplementary Material 1: Additional file, A1 [file 41687_2026_1057_MOESM1_ESM.docx]

**Additional file A1: Rapid umbrella scoping review**

*Methods:* To structure discussions for the workshops, we scoped the existing literature on aspects of palliative and end-of-life care that are important to patients and carers. As several systematic reviews had already been completed in this area, we limited the rapid scoping review to systematic reviews, but not by patient age, condition, country or date. Search terms were developed from review of keywords used in relevant papers and the Cochrane Library (Table A1).

**Table A1:** Search terms (Medline, July 2023)

|  | **Population** | **Intervention** | **Outcome** | **Design** |
| --- | --- | --- | --- | --- |
| **OR** | exp Terminally Ill/ | End of life care.mp. or exp Terminal Care/ | Quality of care.mp. or exp "Quality of Health Care"/ | exp "Systematic Review"/ or exp "Review"/ |
|  | life-limiting condition.mp. | exp Palliative Care/ | quality care.mp. | literature review.mp. |
|  | exp Patients/ | exp Patient Comfort/ | meaningful.mp. |  |
|  | exp Caregivers/ | good death.mp. | exp Patient-Centered Care/ or exp Patient Satisfaction/ |  |
|  | exp Adult/ or exp Family/ | bereavement care.mp. or exp Hospice Care/ | exp "Standard of Care"/ |  |
|  | Children.mp. or exp Child/ |  | preferences.mp. or exp Patient Preference/ |  |
|  | exp Patients/ |  | priorities.mp |  |
|  |  |  | perspectives.mp. |  |
| **AND** | **9,820,589** | **113,528** | **8,997,328** |  |
|  | **39,012** | | | **3,338,494** |
|  | **3,012** | | | |

Searching took place in July 2023 and was limited to the Medline bibliographic database. Screening and data extraction were completed by LW, using a data extraction template including study aim, population, review design, synthesis method, number of studies, included study characteristics (e.g. sample size and setting) and aspects of palliative and end-of-life care that are important to patients and carers. To mitigate the likelihood of underserved populations being under-researched, we supplemented the review with findings from primary studies looking specifically at the influence of ethnicity, sexuality, poverty, and housing on perceived quality of palliative and end-of-life care. No quality assessments were completed.

Findings were synthesised and categorised according to a pre-established framework. Several frameworks were considered, including patient-orientated frameworks, such as Tang *et al.* (2004),^1^ which included the domains spirituality, pain, physical performance and social support; and system-orientated frameworks, such as Zimmerman *et al.* (2019),^2^ which included principles of care (family-centred, patient-led, attentive, flexible) and domains of care (coping and support, symptom control, decision-making, future planning)​. Quality frameworks considered included national quality frameworks for palliative care and rural palliative care,^3^ and the concept of quality of life of dying persons.^4^ However, all these frameworks were considered limited or too complex to accommodate scoping review findings to communicate in an accessible way.

The de Wolf-Linder *et al.* (2019) framework^5^ offered clarity of domains to facilitate information-sharing at PPI workshops. This framework comprises seven most important palliative care outcomes based on international expert consensus. The seven outcome domains included: Overall wellbeing, Physical wellbeing, Emotional wellbeing, Social and family wellbeing, Spiritual wellbeing, Information and preferences and Adverse events and staff distress.^5^

As well as presenting important areas of palliative and end-of-life in an accessible way, the de Wolf-Linder *et al.* (2019) framework also permitted ranking of aspects of care in order of importance and gave opportunity to compare views from PPI members and those from clinical experts. Therefore, we considered this to be a fitting framework for the synthesised findings.

*Results*: We systematically reviewed just over 3,000 papers to understand what areas of palliative and end-of-life care are important to patients and carers, across the lifespan. A total of 23 reviews were included, supplemented with four primary research studies (Figure A1).

**Figure A1:** Flow chart


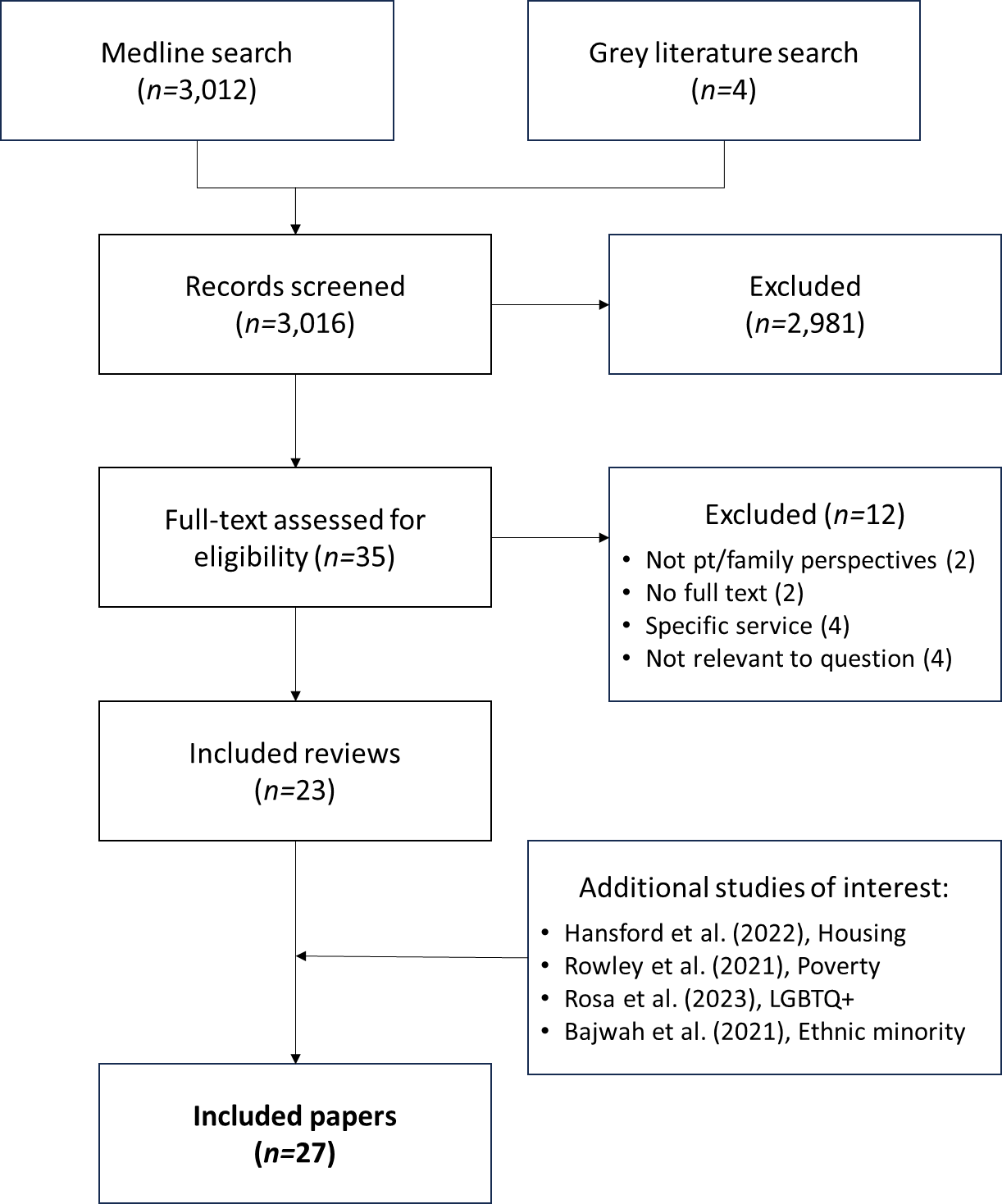


We categorised findings according to the seven de Wolf-Linder domain labels and altered one domain label (‘adverse events and staff distress’) to ‘clinical care and expertise’ and separated ‘social and family wellbeing’ into ‘social wellbeing’ and ‘family wellbeing’.

**References**

1. Tang W-R, Aaronson LS, Forbes SA. Quality of life in hospice patients with terminal illness. Western Journal of Nursing Research. 2004;26(1):113-28.

2. Zimmermann C, Ryan S, Hannon B, Saltman A, Rodin G, Mak E, et al. Team-based outpatient early palliative care: a complex cancer intervention. BMJ supportive & palliative care. 2024;14(e1):e700-e9.

3. Goodridge D, Duggleby W. Using a quality framework to assess rural palliative care. Journal of Palliative Care. 2010;26(3):141-50.

4. Stewart AL, Teno J, Patrick DL, Lynn J. The concept of quality of life of dying persons in the context of health care. Journal of pain and symptom management. 1999;17(2):93-108.

5. de Wolf-Linder S, Dawkins M, Wicks F, Pask S, Eagar K, Evans CJ, et al. Which outcome domains are important in palliative care and when? An international expert consensus workshop, using the nominal group technique. Palliative medicine. 2019;33(8):1058-68.
