# Supplementary material for: Identifying aspects of palliative and end-of-life care that are most important to people with lived experience and can be measured using routine data: a series of patient and public involvement workshops
Source: J Patient Rep Outcomes. 2026 Apr 18;10:120. doi: 10.1186/s41687-026-01057-6 (PMC13369061; doi:10.1186/s41687-026-01057-6)
Supplement: Supplementary file 2 — Supplementary Material 2: Additional file, A2 [file 41687_2026_1057_MOESM2_ESM.docx]

**Additional file A2: Ethical considerations**

Although no ethical approval was needed for this Patient and Public Involvement (PPI) activity,^1^ we adopted an ethical approach, informed by advice and guidance available in the literature,^2, 3^ and other sources.^4^ We have summarised the steps we took to ensure a duty of care during the conduct of the PPI activities, based on the issues outlined in the NIHR Ethical Practice Guidelines for Public Involvement and Community Engagement.^4^

***Safeguarding:*** Both facilitators aimed to create a safe environment online to facilitate discussions. They both had DBS clearance and were aware of internal procedures to escalate any concerns. No safeguarding concerns were raised during the workshops.

***Access, inclusion and equity:*** We shared advertisements for the workshops widely, not only using existing PPI forums, but also through word-of-mouth. To ensure group diversity, we stated in the advert that we were particularly interested in hearing from people from minoritised ethnic groups and people with experience of financial difficulty. While online workshops present barriers for those with limited internet access, we were able to open the workshops up to people from across the country to ensure broad geographical spread.

***Working challenging and sensitive areas of research:*** We were aware that the nature of discussions may have precipitated distress among attendees. For this reason, we developed a distress protocol for use during the workshops, scheduled breaks and encouraged attendees to take breaks as needed and maintained open communication with attendees in between workshops. The distress protocol was not used.

***Confidentiality / consent / assent:*** All information about the workshops were shared with attendees beforehand. The contact details were not shared with others in the group, making use of the ‘bcc’ function on email and emailing attendees individually. Verbal agreement was obtained from attendees to record the workshop discussions and chat for the purpose of enhancing our notes. Recordings were saved to the computer (not external cloud) and confidentially destroyed once no longer needed. Direct quotes were taken from recordings and included in the manuscript with explicit agreement, obtained retrospectively from attendees via email.

***Agreeing boundaries from the outset:*** We discussed details and expectations with attendees prior to the workshops. We agreed with public members of the project team of their involvement in project and their supportive role in the workshops in advance.

***Communication:*** Two facilitate two-way communication, attendees of the workshops were randomly divided into two breakout groups, which were facilitated. We invited comments from all group members and encouraged the use of the ‘raise hand’ function to facilitate communications. We have developed a summary of this work in an accessible format for dissemination. We maintained open communication with public members of the project team, having regular meetings during the project and maintaining contact over email in between meetings.

***Training and support:*** The facilitators had completed relevant internal and external training. Public members of the project team were very experienced in supporting research projects and PPI activities.

***Developing sustainable reciprocal relationships:*** We followed up with attendees to share the manuscript to demonstrate the impact of their contributions. We also invited them to join the departmental PPI forum to remain involved in research engagement activities. We liaised with public members of the project team regularly during the project and ensured their opportunity for contribution on the development and delivery of PPI workshops, analysis and write-up.

***Managing conflict:*** There was no conflict during workshop discussions. Facilitators were experienced in hosting PPI activities and comfortable to swiftly de-escalate tensions should any have arisen.

***Valuing public contributors:*** We followed up with attendees to thank them for their involvement in the workshops. We followed up with attendees to share the manuscript to demonstrate the impact of their contributions. We also ensured that each attendee was reimbursed up to £80.00 after each workshop, as per the NIHR public contributor payment policy.^5^

**References**

1. Nollett C, Eberl M, Fitzgibbon J, Joseph-Williams N, Hatch S. Public involvement and engagement in scientific research and higher education: the only way is ethics? Research Involvement and Engagement. 2024;10(1):50.

2. Suri S, Harrison SL, Bevin-Nicholls A, Shenton F, Atkinson S, Earle J, et al. Patient and public involvement and engagement: Do we need an ‘ethical anchor’? Research Involvement and Engagement. 2024;10(1):113.

3. Austin AM, Carmichael DQ, Bynum JPW, Skinner JS. Measuring racial segregation in health system networks using the dissimilarity index. Social science & medicine (1982). 2019;240:112570.

4. NIHR Applied Research Collaboration North East and North Cumbria. Ethical Practice Guidelines for Public Involvement and Community Engagement 2024 [Available from: <https://arc-nenc.nihr.ac.uk/wp-content/uploads/2024/08/Ethical-Practice-Guidelines-FINAL-July-24.pdf>.

5. National Institute for Health and Care Research. NIHR public contributor payment policy 2022 [Available from: <https://www.nihr.ac.uk/nihr-public-contributor-payment-policy>.
