# Supplementary material for: Identifying aspects of palliative and end-of-life care that are most important to people with lived experience and can be measured using routine data: a series of patient and public involvement workshops
Source: J Patient Rep Outcomes. 2026 Apr 18;10:120. doi: 10.1186/s41687-026-01057-6 (PMC13369061; doi:10.1186/s41687-026-01057-6)
Supplement: Supplementary file 3 — Supplementary Material 3: Additional file, A3 [file 41687_2026_1057_MOESM3_ESM.docx]

**Additional file A3: Guidance for Reporting Involvement of Patients and the Public (GRIPP2)**

| **Section and topic** | **Item** | **Page no.** |
| --- | --- | --- |
| 1: Aim | Report the aim of PPI in the study | 2 |
| 2: Methods | Provide a clear description of the methods used for PPI in the study | 2-4 |
| 3: Study results | Outcomes—Report the results of PPI in the study, including both positive and negative outcomes | 4-7 |
| 4: Discussion and conclusions | Outcomes—Comment on the extent to which PPI influenced the study overall. Describe positive and negative effects | 7-8 |
| 5: Reflections/critical perspective | Comment critically on the study, reflecting on the things that went well and those that did not, so others can learn from this experience | 7-8 |
